# A Survey of Adolescent and Caregiver Perceptions of Substance Use Screening in Pediatric Emergency Departments

**DOI:** 10.1016/j.acepjo.2025.100198

**Published:** 2025-06-07

**Authors:** Stephen Sandelich, Cameron Coakes, Heather L. Stuckey-Peyrot, Sharon Levy, Christopher Buresh, Thomas H. Chun, Aleksandra E. Zgierska

**Affiliations:** 1 Departments of Pediatrics and Emergency Medicine, Penn State College of Medicine, Hershey, Pennsylvania, USA; 2 Penn State College of Medicine, Hershey, Pennsylvania, USA; 3 Department of Medicine, Penn State College of Medicine, Hershey, Pennsylvania, USA; 4 Division of Addiction Medicine, Boston Children's Hospital, Boston, Massachusetts, USA; 5 Department of Emergency Medicine, University of Washington, Seattle, Washington, USA; 6 Department of Pediatrics and Emergency Medicine, Warren Alpert Medical School of Medicine at Brown University, Providence, Rhode Island, USA; 7 Departments of Family and Community Medicine, Public Health Sciences, and Anesthesiology and Perioperative Medicine, Penn State College of Medicine, Hershey, Pennsylvania, USA

**Keywords:** adolescent substance use screening, pediatric emergency department, substance use disorder screening, caregiver perceptions, healthcare screening barriers

## Abstract

**Objectives:**

Substance use among adolescents poses significant health risks; early identification is crucial. Understanding adolescents' and caregivers' perceptions toward substance use screening in emergency departments (EDs) is needed to develop acceptable and effective screening programs.

**Methods:**

A national online survey recruited adolescents aged 13-17 years and caregivers of adolescents via a Qualtrics research panel. The survey included 34 questions for adolescents and 28 for caregivers, assessing attitudes, perceived barriers, and facilitators of ED-based substance use screening through Likert-scale, multiple-choice, and open-ended qualitative responses. Data were analyzed using descriptive statistics, chi-square tests for group comparisons, and thematic analysis for qualitative responses.

**Results:**

Among the 548 adolescent and 544 caregiver respondents, 43.8% of adolescents and 42.6% of caregivers supported universal substance use screening in the ED, whereas 50.4% of adolescents and 38.0% of caregivers rated screening as “very” or “extremely” important. Privacy concerns were identified as a barrier for the majority of adolescents (75.5%) and caregivers (67.8%). Supportive health care staff and clear communication were key facilitators for adolescents (70.0%) and caregivers (74.0%). Adolescents preferred follow-up in the form of digital tools. Thematic analysis of qualitative responses revealed shared concerns among adolescents and caregivers, including privacy, judgment, and test accuracy, whereas adolescents emphasized autonomy and comfort, and caregivers prioritizing transparency, involvement, and legal implications.

**Conclusion:**

Most adolescents and caregivers did not definitely support substance use screening in EDs and identified distinct barriers and facilitators. Addressing concerns about privacy, legal consequences, and trust while leveraging digital tools and other follow-up resources may enhance screening acceptability and reach. Future clinical trials can leverage this information to design and evaluate interventions for optimal substance use screening among adolescents in EDs.

**Bottom Line**

Do adolescents and caregivers support substance use screening in the emergency department? In this national survey of 548 adolescents and 544 caregivers, fewer than half supported universal screening (43.8% of adolescents, 42.6% of caregivers), and only 50.4% of adolescents and 38.0% of caregivers rated it as very or extremely important. Privacy was the top concern (75.5% of adolescents, 67.8% of caregivers). Supportive health care staff and clear communication were key facilitators. Adolescents preferred digital follow-up. Addressing privacy concerns, legal fears, and trust issues may improve screening acceptance and effectiveness in emergency care.

## 1 Introduction

### 1.1 Background

Adolescent substance use disorders (SUDs) pose significant public health risks, increasing morbidity, mortality, and long-term health consequences.^1^ In the United States, approximately 3.8 million adolescents aged 12-17 years reported past-year substance use, and 2.2 million met the criteria for a SUD.^2^ SUDs correlate with risky sexual behaviors, violence, mental health crises, and suicide risk, often leading to emergency department (ED) visits.^3^ Early identification and intervention are critical for mitigating these risks.^4^, ^5^, ^6^ EDs serve as vital access points for adolescents, particularly those who may not engage in routine primary care^6^, ^7^, ^8^, ^9^, and offer an opportunity to proactively address SUD in a timely manner by trusted and knowledgeable staff. Implementing effective SUD screening programs requires an in-depth understanding of adolescents and their caregivers' concerns, preferences, and attitudes.^10^, ^11^, ^12^, ^13^ The Screening, Brief Intervention, and Referral to Treatment (SBIRT) model is widely recommended in pediatric primary care for substance use screening. The American Academy of Pediatrics endorses universal screening for substance use, emphasizing that screening in both primary care and ED settings can enhance early identification and intervention.

SUDs encompass problematic use of alcohol, cannabis, prescription medications, and illicit substances, leading to impairment in functioning.^14^ Among adolescents, cannabis and alcohol are the most commonly used substances, followed by nicotine, prescription opioids, and stimulants.^1^^,^^2^ The unique nature of ED encounters presents both challenges and opportunities for SUD screening.^15^^,^^16^ Adolescents and families are vulnerable to stigma, judgment, and concerns about loss of confidentiality that the fast-paced ED environment can exacerbate.^17^^,^^18^ Similarly, caregivers may have concerns about the accuracy, purpose, and outcomes, including legal implications, of SUD screenings for their children.^19^ Caregivers and patients may not understand how SUD screening is related to their reason for visiting. Addressing these concerns is essential for fostering trust and participation.

Prior research highlights the prevalence of adolescent substance use and the ED’s unique role in identifying and addressing risky behaviors. Renny et al^13^ surveyed adolescents in the emergency department and found that 71.8% of adolescents in their cohort thought that it was important or very important to ask teens about alcohol and drug use in the ED. Mello et al^12^ evaluated adolescents’ perceptions of SBIRT in pediatric trauma centers and found that although implementation improved documentation rates, adolescents perceived being asked about substance use more frequently compared with that recorded in electronic health records, indicating gaps in implementation fidelity and patient experience.

Beyond acceptability, Langerman et al^10^ assessed attitudes toward ED-based social and behavioral health screening, finding that 84% of caregivers and 62% of adolescents supported substance use screening in the ED. However, these rates suggest a discrepancy between adolescent and caregiver perspectives, which could influence screening uptake and intervention effectiveness. Additionally, O’Mara et al^11^ examined screening for suicide risk and mental health conditions in the ED, finding that parents were more supportive of screening than adolescents, particularly regarding routine implementation. This discrepancy underscores the need for research exploring both adolescent and caregiver perceptions, particularly in the context of universal substance use screening.

### 1.2 Importance

Although these studies highlight the importance of ED-based screening, they primarily focus on individual substances (eg, cannabis or alcohol) or specific screening models, such as SBIRT.^10^^,^^11^ Our study builds on this literature by examining perceptions of universal substance use screening across a broad range of substances, independent of SBIRT or trauma-specific interventions. Furthermore, unlike prior studies that engaged caregivers and adolescents in screening design, our study assessed attitudes toward an externally developed screening process. By addressing these gaps, our findings contribute novel insights into optimizing ED-based substance use screening protocols to improve adolescent engagement and intervention effectiveness.

### 1.3 Goals of This Investigation

This study explored adolescent and caregiver perspectives and priorities regarding SUD screening of adolescents. The goal was to inform tailored, acceptable, and effective SUD screening programs in EDs and, ultimately, reduce adolescent SUD-related harms.

## 2 Methods

### 2.1 Study Design

This is a cross-sectional survey study design and follows the Consensus-Based Checklist for Reporting of Survey Studies reporting guidelines.^20^

### 2.2 Setting

Invitations and links to the online surveys were distributed by Qualtrics.

### 2.3 Selection of Participants

Surveys were sent to potential participants via email in 2 waves. The first pilot wave (October 3, 2024 to October 7, 2024) collected data from 50 adolescents and 50 caregivers (∼10% of the predicted total sample size of 500 per group) to enable the evaluation of internal consistency of the survey’s questions; the collected responses yielded Cronbach alpha values of 0.917 and 0.714, respectively, indicating good internal consistency. A second wave of surveys was distributed between October 9, 2024 and October 17, 2024 to reach the targeted sample size.

The research team recruited participants through a national pool of research respondents from across the United States maintained by the Qualtrics platform. Potential participants were identified from Qualtrics' existing panel members who met the study’s age and caregiver eligibility criteria. English-fluent adolescents aged 13-17 years and caregivers of children aged 13-17 years were eligible for participation. The adolescent and caregiver samples were recruited separately (ie, they did not represent child-caregiver dyads). Invitations were sent via email, informing participants that the survey aimed to assess perceptions of adolescent substance use screening in emergency departments (EDs). No further details about specific survey questions or outcomes were disclosed prior to participation. Participants were compensated for their survey completion through the Qualtrics panel with a small monetary incentive in the form of gift cards, gash, airline miles, or vouchers. The research team received deidentified, participant-level data from Qualtrics.

The target sample size of approximately 500 respondents per group was determined based on feasibility, logistical constraints, and a review of past literature using similar survey methodologies. This number was selected to provide a sufficient sample for meaningful subgroup analyses while maintaining practical feasibility within the recruitment timeline and budget constraints.

The institutional review board reviewed and approved the study protocol as exempt. Survey initiation was considered an implied consent to participate in the study. No identifiable information was collected or linked to the analyzed survey responses.

### 2.4 Interventions

A panel of experts from the Pediatric Emergency Care Applied Research Network (PECARN) SUD Interest Group contributed to the design and content of adolescent and caregiver surveys. Survey validation involved interviews with individual adolescents (n = 9) and caregivers (n = 10) to ensure the questions’ clarity, relevance, and nonstigmatizing language. A group of trained adolescent stakeholders (n = 4) from the Penn State Clinical and Translational Science Institute supported the Adolescent Health Network, further refined the adolescent survey content.^21^

Qualitative responses were solicited from adolescents and caregivers using 4 questions (Qs), including (Q1) concerns related to adolescents are screened for substance use; (Q2) what would make adolescents/caregivers feel more comfortable during the screening process; (Q3) suggestions to improve adolescent SUD screening; and (Q4) adolescent/caregiver perceptions on how ED doctors can better support adolescents and their families during the screening process.

### 2.5 Measurements

The finalized surveys (see Supplemental Material) included 34 questions (30 quantitative, 4 qualitative) for adolescents and 28 questions (24 quantitative, 4 qualitative) for caregivers to gather data on attitudes, perceptions, barriers, and facilitators toward SUD screening in EDs. Overall, the caregiver’s survey mirrored the content of the adolescent version but centered on caregivers' perspectives regarding their child’s screening. Some questions in the adolescent survey were not asked of caregivers (see Surveys in the Supplemental Material). Quantitative responses primarily offered a 0-4-point Likert scale (0=never; 4=always). Demographic data were collected via self-report, following the existing guidelines.^22^ This study focused on adolescent and caregiver perceptions of questionnaire-based screening rather than biological tests such as urine toxicology screens, which may introduce additional concerns regarding accuracy and privacy.

### 2.6 Data Analysis

Qualitative thematic analysis of responses to 4 qualitative questions answered by adolescents and caregivers was performed using MAXQDA software version 24 (VERBI Software), with the analytical codebook developed through inductive coding. Two coders (S and CC) applied the codes to a random selection of 20% of the dataset to establish interrater reliability, which resulted in Cohen *κ* = 0.94. A third coder (TW) reviewed coding discrepancies and adjudicated disagreements. The coding was finalized via a consensus approach; all disagreements were resolved.

Quantitative data were analyzed using SAS statistical software version 9.4 (SAS Institute Inc.). Unless stated otherwise, descriptive statistics (means ± SD or frequencies) summarized participant demographics and quantitative responses. Inferential statistics, including chi-square tests or t tests, assessed differences in perceptions and attitudes across demographic groups (eg, age, gender); a statistical significance level was set at a two-tailed *P* < .05.

## 3 Results

### 3.1 Survey Participants

Invitation emails with a survey link were sent to 3,234 adolescents and 3,525 caregivers. The survey was completed by 548 adolescents and 544 caregivers (17.0% and 15.4% response rates, respectively) who formed the study sample and were included in the analysis. Study completion took 6.1 ± 5.4 minutes for adolescents and 7.0 ± 7.7 minutes for caregivers.

### 3.2 Sample Characteristics

The adolescent participants (N = 548) were 15.5 ± 1.7 years, with 50.4% identifying as male. The majority were White/Caucasian (56.6%), followed by Black/African American (24.8%), Hispanic/Latino (10.4%), and Asian (4.0%). Most adolescents reported living with 2 caregivers (52.5%); others reported living with mother only (21.0%), father only (8.03%), or in other arrangements, such as independent living (5.1%) (Table 1).

**Table 1:** Characteristics of the adolescent (N = 548) and caregiver (N = 544) samples.

Variable	Adolescents (N = 548)	Caregivers (N = 544)
Age		
Adolescents: y, mean (SD)	15.5 (1.7)	
Caregivers: range, y, n (%):	-	
20-29		7 (1.2)
30-39	-	131 (24.1)
40-49	-	276 (50.7)
50-59	-	98 (18.0)
60-69	-	24 (4.4)
70-79	-	8 (1.5)
Gender, n (%)		
Male	276 (50.4)	275 (50.6)
Female	269 (49.1)	269 (49.5)
Other	3 (0.6)	0 (0.0)
Ethnicity, n (%)		
Asian	22 (4.0)	13 (2.4)
Black/African American	136 (24.8)	107 (19.7)
Hispanic/Latino	57 (10.4)	38 (7.0)
Middle Eastern/North African	1 (0.2)	0 (0.0)
Multiracial/multiethnic	13 (2.4)	7 (1.3)
Native American/Alaska Native	6 (1.1)	11 (2.0)
Native Hawaiian/Pacific Islander	3 (0.6)	366 (67.3)
White/Caucasian	310 (56.6)	2 (0.4)
Living situation, n (%)		
With both parents	288 (52.6)	-
With mother only	115 (20.1)	-
With father only	44 (8.0)	-
Independent living (eg, alone or with roommates)	28 (5.1)	-
Joint custody/shared between parents	26 (4.7)	-
With grandparents	9 (1.6)	-
With other relatives	4 (0.7)	-
In a boarding school or residential school	3 (0.6)	-
With foster parents	2 (0.4)	-
Prefer not to say/other	29 (5.3)	-
Highest level of completed education, n (%)		
Less than high school	-	34 (6.3)
High school graduate	-	149 (27.4)
Some college	-	118 (21.7)
2-y degree	-	47 (8.6)
4-y degree	-	81 (14.9)
Professional degree	-	105 (19.3)
Doctorate	-	10 (1.8)

Among the caregiver participants (N = 544), the majority reported being 40–49 years (50.7%) and identified as male (50.6%) and White/Caucasian (67.3%), followed by Black/African American (19.7%), Hispanic/Latino (7.0%), and Asian (2.4%). Close to one-half (49.1%) reported up to high school-level education; smaller proportions reported having college (23.5%) or professional (19.3%) degrees.

### 3.3 Adolescent and Caregiver Experiences with SUD Screening

Among adolescents (N = 548), the majority (79.0%) reported prior personal ED visits, whereas 81.6% of 544 caregiver-respondents reported prior ED visits for their child, with many in both groups noting multiple prior visits. In adolescents and caregivers who reported prior ED visits (433 adolescents, 444 caregivers), the minority recalled receiving (144 and 62, respectively) SUD screening for themselves (adolescents) or their child (caregivers) during their ED visit, with 36 and 42, respectively, being unsure if they received it (Table 2).

**Table 2:** Adolescent and caregiver experiences with substance use disorder (SUD) screening.

Survey question, n (%)	Adolescents			Caregivers		
Have you ever visited the ED [for your child]?	(N = 548)			(N = 544)		
Yes	416 (75.9)			441 (81.0)		
No	115 (21.0)			100 (18.3)		
Not sure	17 (3.1)			3 (0.6)		
*All participants who answered “Yes” or “Not Sure”: Have you ever visited the emergency department [for your child]?*	(N = 433)			(N = 444)		
How many times have you visited the ED [for your child]?	(N = 433)			(N = 444)		
1	107 (24.7)			113 (25.4)		
2-3	223 (51.5)			228 (51.4)		
≥4	103 (23.8)			103 (23.2)		
Have you [your child] ever been screened for substance use in the ED?	(N = 433)			(N = 444)		
Yes	141 (32.6)			62 (14.0)		
Not sure	36 (8.3)			42 (9.4)		
No	256 (59.1)			340 (76.6)		
How many times have you [your child] been screened for drug use in the ED?	(N = 177)			(N = 104)		
Never	14 (7.9)			3 (2.9)		
Once	75 (42.4)			52 (50.0)		
≥Twice	59 (33.3)			34 (32.7)		
Not sure	29 (16.4)			15 (14.4)		
Was your parent ever present for your drug use screening in the ED [Have you ever been present during your child's drug use screening in the ED]?	Screened = Yes (N = 141)	Screened = Unsure (N = 36)	*P* value (*χ*^*2*^)	Screened = Yes (N =) (N = 62)	Screened = Unsure (N = 42)	*P* value (*χ*^*2*^)
Yes	124 (88.0)	21 (58.3)	0.0016	58 (93.5)	25 (59.5)	0.0004
No	14 (9.9)	8 (22.2)		4 (6.4)	8 (19.0)	
Not sure	3 (2.13)	7 (17.4)		0 (0)	9 (21.4)	
How comfortable did you feel [think your child felt] during the screening process?	(N = 141)	(N = 36)		(N = 62)	(N = 42)	
Extremely uncomfortable	24 (17.0)	2 (5.6)	0.0041	9 (14.5)	5 (11.9)	0.0228
Somewhat uncomfortable	30 (21.3)	7 (19.4)		11 (17.7)	7 (16.7)	
Neither comfortable nor uncomfortable	18 (12.8)	17 (47.2)		4 (6.5)	13 (30.9)	
Somewhat comfortable	28 (19.9)	6 (16.6)		19 (30.6)	9 (21.4)	
Extremely comfortable	41 (29.1)	4 (11.1)		19 (30.6)	8 (19.0)	
How did you feel about the length of the screening process?	(N = 141)	(N = 36)		(N = 62)	(N = 42)	
Extremely inappropriate	8 (5.7)	2 (5.6)	0.0187	2 (3.2)	1 (2.3)	0.0481
Somewhat inappropriate	13 (9.2)	1 (2.8)		2 (3.2)	5 (11.9)	
Neither appropriate or inappropriate	20 (17.2)	12 (33.3)		7 (11.3)	8 19.0)	
Somewhat appropriate	44 (31.2)	11 (30.6)		22 (35.5)	15 (35.7)	
Extremely appropriate	56 (39.7)	10 (27.8)		29 (46.8)	13 (30.9)	
Do you think the screening process took too much time during your [your child’s] ED visit?	(N = 141)	(N = 36)		(N = 62)	(N = 42)	
Definitely not	20 (14.2)	2 (5.6)	0.0295	10 (6.2)	6 (14.3)	0.0753
Probably not	27 (19.1)	1 (2.7)		20 (31.2)	11 (26.2)	
Might or might not	23 (16.3)	12 (33.3)		15 (24.2)	15 (35.7)	
Probably yes	38 (26.9)	11 (30.6)		7 (11.3)	3 (7.1)	
Definitely yes	33 (23.4)	10 (27.7)		10 (6.2)	7 (16.7)	
Did you feel the screening process was respectful?	(N = 141)	(N = 36)		(N/A)	(N/A)	
Definitely not	9 (6.4)	1 (2.7)	0.0441			
Probably not	9 (6.4)	4 (11.1)				
Might or might not	27 (19.1)	12 (33.3)				
Probably yes	50 (35.5)	11 (30.6)				
Definitely yes	46 (32.6)	8 (22.2)				
Were the screening questions easy to understand?	(N = 141)	(N = 36)		(N/A)	(N/A)	
Yes	119 (84.4)	30 (83.3)	0.5894			
No	6 (4.2)	1 (2.8)				
Maybe	16 (11.3)	5 (13.9)				
Were there any questions you found difficult to answer?	(N = 141)	(N = 36)		(N/A)	(N/A)	
Yes	26 (18.4)	2 (5.6)	0.1512			
No	91 (64.5)	30 (83.3)				
Maybe	24 (17.0)	4 (11.1)				
Did the screening process help you understand the risks of substance use better?	(*N* = 141)	(N = 36)		(N/A)	(N/A)	
Definitely not	6 (4.3)	2 (5.6)	0.0316			
Probably not	11 (7.8)	7 (19.4)				
Might or might not	22 (15.6)	11 (30.6)				
Probably yes	53 (37.6)	11 (30.6)				
Definitely yes	49 (34.8)	5 (13.9)				

ED, emergency department.

Among those who received or potentially received SUD screening (144 adolescents, 62 caregivers), most adolescents (88.0%) and caregivers (93.5%) reported that a parent/caregiver was present during the screening. They expressed varied comfort levels with the SUD screening process, with 49.0% of adolescents and 61.2% of caregivers reporting feeling comfortable (“extremely” or “somewhat”) with this screening. Regarding the screening process length, 70.9% of adolescents and 83.3% of caregivers found its length appropriate (“extremely” or “somewhat”).

For adolescents who experienced SUD screening in the ED (N = 141), most (84.4%) found the screening questions easy to understand, with 64.5% reporting that no questions were difficult to answer. Most of them (68.1%) felt respected during the screening process (“definitely” or “probably”), and 72.4% indicated that it helped (“definitely” or “probably") improve their understanding of substance use−related risk.

Adolescents who definitively recalled SUD screening (N = 141) were significantly more likely than those who were unsure (N = 36) to perceive the process as respectful (68.1% vs 52.8%, *P* = .044), comfortable (48.8% vs 27.7%, *P* = .004), and appropriate in length (70.9% vs 58.4%, *P* = .019). They were also more likely to report that a parent was present during screening (88.0% vs 58.3%, *P* = .002) and that the screening helped them understand the risks of substance use (72.4% vs 44.5%, *P* = .032). Similarly, caregivers who recalled screening (N = 62) were significantly more likely than those who were unsure (N = 42) to report being present during their child’s screening (93.5% vs 59.5%, *P* < .001), to feel comfortable with the screening process (61.2% vs 40.4%, *P* = .023) and to perceive the screening as appropriate in length (*P* = .048).

### 3.4 Adolescent and Caregiver Attitudes and Perceptions of SUD Screening

#### 3.4.1 Importance of screening

All adolescents (N = 548) and caregivers (N = 544), regardless of their prior history of experiencing SUD screening in the ED, were asked about their attitudes and perceptions of screening. One-half of adolescents (50.4%) viewed SUD screening in the ED as “very” or “extremely” important, while only 38.0% of caregivers shared those views. In our cohort, 43.8% of adolescents and 42.6% of caregivers believed that SUD screening should be a routine part of all adolescent ED visits, whereas 19.2% of adolescents and 38.2% of caregivers believed that it should not be conducted routinely (Table 3).

**Table 3:** Adolescent (N = 548) and caregiver (N = 544) attitudes and perceptions of substance use disorderscreening in the emergency department.

Survey question, n (%)	Adolescents (N = 548)	Caregivers (N = 544)
How important do you think it is for adolescents to be screened for drug use in the emergency department?		
Not at all important	37 (6.8)	80 (14.7)
Slightly important	94 (17.2)	131 (24.1)
Moderately important	141 (25.7)	126 (23.2)
Very important	145 (26.5)	115 (21.1)
Extremely important	131 (23.9)	92 (16.9)
Do you believe screening for drug use should be a routine part of emergency department visits?		
Yes	240 (43.8)	232 (42.6)
No	105 (19.2)	208 (38.2)
Maybe	175 (31.9)	78 (14.3)
Not sure	28 (5.1)	26 (4.8)
Do you think drug use screening in the emergency department can help teens?		
Definitely not	15 (2.7)	24 (4.4)
Probably not	52 (9.5)	72 (13.3)
Might or might not	131 (23.9)	37 (6.8)
Probably yes	184 (33.6)	229 (42.2
Definitely yes	166 (30.3)	181 (33.3)
Do you think screening for drug use can help prevent drug use in your friends?		N/A
Definitely not	28 (5.1)	
Probably not	84 (15.3)	
Might or might not	150 (27.4)	
Probably yes	149 (27.2)	
Definitely yes	137 (25.0)	
How do you feel about being asked questions about drug use/How do you feel about doctors asking your child about their drug use?		
Extremely uncomfortable	34 (6.2)	40 (7.4)
Somewhat uncomfortable	106 (19.3)	65 (11.9)
Neither comfortable nor uncomfortable	129 (23.5)	141 (25.9)
Somewhat comfortable	140 (25.6)	147 (27.0)
Extremely comfortable	139 (25.4)	151 (27.8)
How comfortable would you be about discussing your [your child's] drug use with doctors in the emergency department?		
Extremely uncomfortable	44 (8.0)	20 (3.7)
Somewhat uncomfortable	98 (17.9)	34 (6.3)
Neither comfortable nor uncomfortable	113 (20.6)	102 (18.8)
Somewhat comfortable	123 (22.5)	124 (22.8)
Extremely comfortable	170 (31.0)	264 (48.5)
How likely are you to be honest about your drug use in the emergency department/How likely are you to encourage your child to be honest during drug use screening?		
Extremely unlikely	26 (4.8)	19 (3.5)
Somewhat unlikely	54 (9.8)	11 (2.0)
Neither likely nor unlikely	78 (14.2)	49 (9.0)
Somewhat likely	134 (24.5)	94 (17.3)
Extremely likely	256 (46.7)	371 (68.2)
Do you think the results of the screening should be shared with your parents/guardians? Do you think the results of your child’s screening should be shared with you?		
Definitely not	39 (7.1)	53 (9.7)
Probably not	55 (10.0)	28 (5.1)
Might or might not	82 (15.0)	67 (12..3)
Probably yes	180 (32.9)	168 (30.9)
Definitely yes	192 (35.0)	228 (42.0)
Do you think the results of the screening should be shared with your school counselor?		
Definitely not	127 (23.2)	139 (25.5)
Probably not	99 (18.1)	70 (12.9)
Might or might not	133 (24.3)	196 (36.0)
Probably yes	99 (18.1)	76 (14.0)
Definitely yes	90 (16.4)	63 (11.6)
How likely are you to seek help if you [your child] have substance use issues?		
Extremely unlikely	22 (4.0)	18 (3.3)
Somewhat unlikely	40 (7.3)	15 (2.8)
Neither likely nor unlikely	101 (18.4)	39 (7.1)
Somewhat likely	181 (33.0)	84 (15.4)
Extremely likely	204 (37.2)	388 (71.3)
How likely are you to talk to a health care provider about substance use in the future [for your child]?		
Extremely unlikely	44 (8.0)	43 (7.9)
Somewhat unlikely	57 (10.4)	22 (4.0)
Neither likely nor unlikely	149 (27.2)	111 (20.4)
Somewhat likely	163 (29.7)	156 (28.7)
Extremely likely	135 (24.6)	212 (39.0)
Do you think the emergency department is a good place to get information about substance use?		
Definitely not	27 (4.9)	32 (5.9)
Probably not	40 (7.3)	63 (11.6)
Might or might not	142 (25.9)	230 (42.3)
Probably yes	172 (31.4)	114 (21.0)
Definitely yes	167 (30.5)	105 (19.3)

ED, emergency department; SUD, substance use disorder.

#### 3.4.2 Effectiveness of and comfort with screening

Among the surveyed participants, 63.9% of adolescents (N = 558) and 75.5% of caregivers (N = 544) believed that SUD screening in the ED would benefit teens (“probably” or “definitely”). Adolescents were evenly split on whether SUD screening could help prevent substance use among their friends, with 52.2% stating it could help their peers (could “probably” or “definitely”); caregiver perceptions on this topic were not assessed. When asked about their comfort levels with being screened for substance use in the ED, 51.0% of adolescents and 54.8% of caregivers reported feeling comfortable (“extremely” or “somewhat”).

#### 3.4.3 Honesty and transparency in screening

Among adolescents (N = 548), 71.2% reported that if asked about substance use, they were likely (“extremely” or “somewhat”) to respond honestly. Caregivers (N = 544, 84.0%) echoed this sentiment, stating they would be likely (“extremely” or “somewhat”) to encourage their child to answer SUD screening questions honestly. Most adolescents supported transparency in sharing screening results, with 68.0% believing results should “definitely” or “probably” be shared with caregivers. Caregivers supported sharing results, with 72.9% favoring disclosing results to them. Only 34.5% of adolescents and 25.6% of caregivers supported sharing such results with school counselors.

#### 3.4.4 Intentions after screening

Among adolescents (N = 548), 70.2% indicated a high likelihood (“extremely” or “somewhat”) of seeking help for substance use after a positive screen in the ED. Caregivers were even more likely (86.7%) to report they were likely (“extremely” or “somewhat”) to encourage their child to seek help. Over one-half of adolescents (54.3%) and caregivers (67.7%) reported being likely (“extremely” or “somewhat”) to talk to a health care provider about their own/their child’s substance use. Adolescents viewed the ED as a valuable source for substance use−related information, with 61.9% stating the ED was a good place (“definitely” or “probably”) for such information. Caregivers were slightly less enthusiastic, with 40.3% viewing the ED as a good information resource (“definitely” or “probably”).

### 3.5 Potential Barriers to Screening

**Figure 1:**
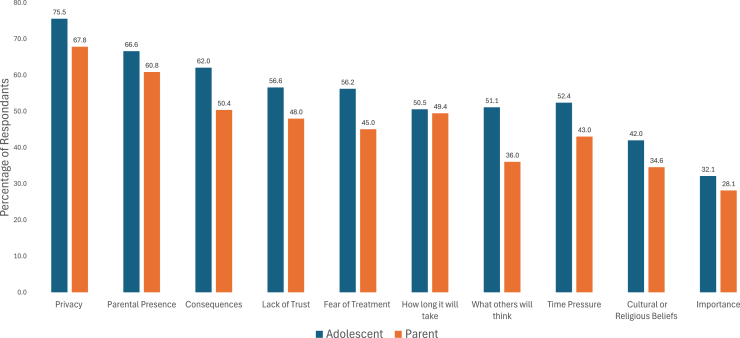
Barriers to substance use screening in the emergency department.

Figure 1 highlights the key barriers to substance use screening as reported by adolescents (N = 558) and caregivers (N = 544). Privacy concerns were the most frequently cited, reported by 75.5% of adolescents and 67.8% of caregivers. The presence of caregivers during screening was also a significant concern, identified as a barrier by 66.6% of adolescents and 60.8% of caregivers. Sixty-two percent of adolescents and 50.4% of caregivers noted fear of legal consequences following a positive screen. At the same time, 56.6% of adolescents and 48.0% of caregivers reported a lack of trust in health care staff. Concerns about the duration of the screening process were expressed by 52.4% of adolescents and 43.0% of caregivers. In comparison, the fear of being forced into treatment following a positive screen was identified by 56.2% of adolescents and 45.0% of caregivers.

Additional barriers included concerns about what others might think (51.1% of adolescents and 36.0% of caregivers), cultural or religious beliefs (42.0% of adolescents and 34.6% of caregivers), and general time pressure (reported by 43.0% of caregivers). Adolescents and caregivers both emphasized the importance of screenings being efficient, private, and nonjudgmental.

### 3.6 Next Steps

**Figure 2:**
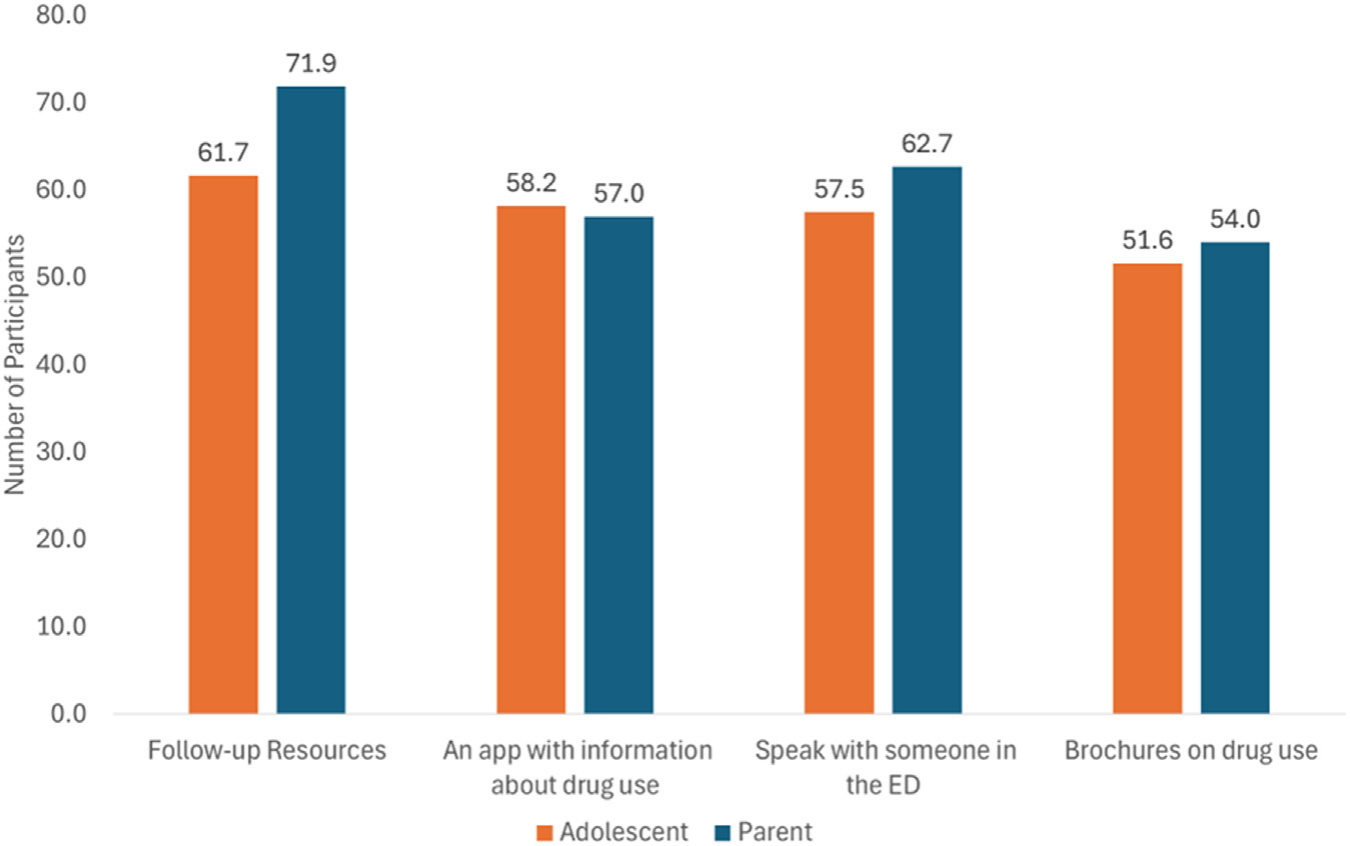
Follow-up interventions. ED, emergency department.

Guidance on the next steps in the form of follow-up resources was the most frequently cited need, selected by 61.7% of adolescents and 71.0% of caregivers. The availability of an app with information about drug use and treatment options was highlighted as a potential facilitator by 58.2% of adolescents and 57.0% of caregivers. The opportunity to speak with an ED professional after a positive screen was preferred by 57.5% of adolescents and 62.7% of caregivers. Brochures on drug use were seen as helpful by 51.6% of adolescents and 54.0% of caregivers (Fig 2).

### 3.7 Qualitative Findings

Thematic analysis of adolescent and caregiver responses to 4 qualitative questions identified distinct themes for each question, described in Table 4, along with representative quotes for each theme.

**Table 4:** Themes in qualitative responses of adolescent (N = 548) and caregiver (N = 544) participants, with representative quotes for each theme.

Q1: What concerns do you have about being screened for drug use?
Theme 1: Privacy and Confidentiality A: Worries about sharing personal information without consent. • *“I am concerned about who has access to the information and what will be done with it.”* • *“It may violate my privacy.”* C: Concerns about confidentiality and unauthorized sharing of results with schools or authorities. • *“Keep everything private.”* • *“I worry about the results being shared with schools or authorities.”*
Theme 2: Fear of Judgment or Stigma A: Concerns about being unfairly judged or labeled as drug users. • “People judging me.” • “Being labeled for life as an addict.” C: Anxiety about the child being unfairly labeled or judged, increasing stigma. • “I worry that my child will be labeled unfairly.” • “Screening could cause shame or stigma if not handled carefully.”
Theme 3: Concerns About Consequences A: Fears of punishment or legal trouble from positive results. • “Getting in trouble at home.” • “I don’t want to be screened and go to jail for using drugs.” C: Concerns about potential legal trouble or social fallout for the child. • “What if my child gets in trouble with the law?” • “I’m worried about how the results could affect their future.”
Theme 4: Test Accuracy and Reliability Issues A: Anxiety about false positives or test errors. • “False positive for something.” • “What if I take certain medications that show up as drugs in my test?” C: Fear of false positives and the impact of inaccurate testing on the child. • “What if the test shows a false positive?” • “Accuracy is important—we can’t afford mistakes.”
Theme 5: Concerns About Necessity or Relevance A: Feeling that screening is unnecessary if they do not use substances. • “I don’t do drugs, so it seems invasive.” • “If I don't use drugs, why do I need a drug test?” C: Questioning the need for screening without signs of substance use. • “Only screen if there are signs of drug use.” • “It shouldn’t be routine without a valid reason.”
Theme 6: Discomfort with the Screening Process A: Embarrassment or discomfort, especially in front of others. • “It’s just embarrassing because I don’t do drugs.” • “It could be uncomfortable to do in front of parents.”
Theme 7: Emotional and Psychological Impact on the Child C: Worries about emotional distress, embarrassment, or rebellion caused by screening. • “It could make my child feel embarrassed.” • “I worry that it might make my child rebel.”
Q2: What would make you feel more comfortable during the screening process?
Theme 1: Privacy and Confidentiality A: Importance of keeping results private and only shared with medical staff. • “If it was done in private.” • “Knowing the results would not be shared with anyone.” C: Desire for confidential handling of results and limited sharing. • “Keep the results confidential and private.” • “Ensure no information is shared without my consent.”
Theme 2: Clear Communication and Transparency A: Need for understanding the purpose and outcomes of the screening. • “Clear communication about what I am being screened for.” • “Knowing exactly why the screening is being done.” C: Need for clear explanations about the process, purpose, and outcomes. • “Explain the reason for screening and what will happen with the results.” • “Transparent communication would make me feel more at ease.”
Theme 3: Supportive and Nonjudgmental Environment A: Preference for health care staff to be empathetic and nonjudgmental. • “Not being judged.” • “A friendly doctor who cares about me.” C: Importance of a nonjudgmental, safe environment during screening. • “Doctors should be supportive, not judgmental.” • “Create an environment where my child feels safe and respected.”
Theme 4: Supportive Presence A: Desire for a familiar or trusted person to be present. “Having my parents with me.” “A friend present.”
Theme 5: Comfortable Environment A: Suggestions for a calm, welcoming environment with small comforts. • “Snacks or drinks would help.” • “Having a TV to watch while waiting.”
Theme 6: Respect and Empowerment A: Desire to be involved in decisions and treated with respect. • “Asking my permission before sharing any information.” • “Being respected and listened to.”
Theme 7: Involvement and Consent Preference for parental involvement and consent for screening. • “Let me be there with my child during the screening.” • “Make sure parents give consent before anything happens.”
Theme 8: Faster and Less Intrusive Process C: Suggestions for a faster, streamlined screening process. • “Make the process faster and easier.” • “A streamlined process with quick results would help.”
Theme C9: Assurance of Accurate Testing Methods C: Emphasis on reliable testing to avoid false positives. • “Ensure the tests are accurate and reliable.” • “Avoid false positives that could cause unnecessary stress.”
Q3: What suggestions do you have to improve the drug screening process?
Theme 1: Privacy and Confidentiality A: Ensuring results are kept confidential and not shared without consent. • “Keep it between me and the doctor.” “Ensure confidentiality, don’t tell parents or police.” C: Ensuring results remain confidential and limiting involvement of external parties • “Keep everything private.” • “Parents may worry about who has access to the screening results.”
Theme 2: Clear Communication and Transparency A: Requests for clear explanations about the screening process. • “Provide clear communication about the process.” • “Explain what happens with the results.” C: Request for clear explanations and educational materials on substance use • “Explain everything clearly.” • “Provide educational sessions on substance use and the importance of screening.”
Theme 3: Nonjudgmental and Supportive Environment A: Need for respectful, nonjudgmental interactions with healthcare staff. • “Make it judgment-free.” • “Don't treat people like drug users.” C: Focus on support rather than punishment to encourage openness. • “Doctors should create an environment where adolescents feel comfortable and not judged.” • “Offer a supportive, non-punitive atmosphere.”
Theme 4: Faster and Less Intrusive Process A: Desire for a quick and efficient screening process. • “Make it quick and easy.” • “Explore less invasive testing methods.” C: Recommendations for a quicker, simpler screening process • “Make the screening faster and less invasive.” • “Explore simpler and quicker ways to screen.”
Theme 5: Follow-Up Support and Resources A: Requests for additional counseling or resources if needed. • “Provide counseling options after screening.” • “Help those with drug problems rather than punish them.” C: Suggestions for follow-up care, counseling, and referrals. • “Offer counseling or guidance services after screening.” • “Provide resources and referrals for further support.”
Theme 6: Comfortable Environment A: Suggestions for a calm setting and small comforts • “Offer snacks and drinks.” • “Provide a welcoming environment with friendly staff.”
Theme 7: Respect and Empowerment A: Desire for control over the process and informed consent. • “Ask for my permission first.” • “Make it my choice to do the screening.”
Theme 8: Involvement and Consent C: Emphasis on involving parents in the process with consent and transparency. • “Parents should be informed and give consent.” • “Talk with parents to make sure it’s OK.”
Theme 9: Cultural Sensitivity and Personalization C: Recommendations for culturally sensitive and individualized screening. • “Use culturally sensitive screening tools.” • “Tailor questions to the individual needs of the child.”
Q4: How can doctors better support adolescents during drug use screening?
Theme 1: Privacy and Confidentiality A: Importance of keeping results confidential and private. • “Keep it between me and the doctor.” • “Make sure the information stays private.” C: Emphasis on ensuring privacy and limiting information sharing with external entities. • “Ensure privacy, don’t share information without consent.” • “Keep screening between the child and the doctor, no involvement from law enforcement.”
Theme 2: Clear Communication and Transparency A: Request for education on the risks of substance use and the purpose of screening. • “Explain why the screening is needed and how it helps.” • “Tell us the health effects of drugs.” C: Request for a nonjudgmental, compassionate tone to foster trust. • “Explain what’s being done and why it’s important.” • “Be transparent about what happens with the results.”
Theme 3: Nonjudgmental and Supportive Environment A: Desire for health care staff to be supportive and nonjudgmental. • “Don’t judge or look down on us.” • “Be understanding and kind, not critical.” C: Request for a nonjudgmental, compassionate tone to foster trust. • “Be non-judgmental and understanding.” • “Treat it as a health issue, not a moral failing.”
Theme 4: Follow-Up Support and Resources A: Suggestions for follow-up counseling or guidance. • “Offer counseling if needed.” • “Give us advice and help with quitting if we need it.” C: Emphasis on offering follow-up care, resources, and guidance. • “Provide referrals and resources to help if the screening is positive.” • “Offer follow-up care and guidance.”
Theme 5: Respect and Empowerment A: Need for respectful, age-appropriate communication • “Talk to us in a way we understand, not with medical jargon.” • “Be respectful and listen to us.”
Theme 6: Involving Parents Thoughtfully A: Desire for private conversations with doctors, with optional parental involvement. • “Let me talk to the doctor alone if I need to.” • “Balance privacy with involving parents when appropriate.”
Theme 7: Involvement and Consent C: Preference for collaborative involvement of parents while respecting adolescent privacy. • “Balance privacy with parental involvement.” • “Work with the family to develop a plan.”
Theme 8: Use of Age-Appropriate Language and Tools C: Recommendations for age-appropriate communication and tools for adolescents • “Use language they can understand.” • “Tailor the process to the adolescent’s age.”
Theme 9: Normalize Screening and Reduce Stigma C: Suggestion to treat screening as a routine healthcare activity to reduce stigma. • “Treat it like a routine part of healthcare.” • “Normalize screening to remove stigma.”

When asked about potential concerns regarding adolescent screening in the ED for substance use, responses of adolescents yielded 6 themes, and those of caregivers yielded 6 themes. Both groups expressed concerns regarding “Privacy and confidentiality,” “Judgement or stigma,” “Concern with consequences,” “Test accuracy and reliability,” and the “Necessity or relevance of screening.” Distinct themes also emerged. Adolescents expressed fears about “Discomfort with the screening process,” whereas caregivers highlighted the “Emotional and psychological impact of screening” on their children.

When asked about what would make them feel more comfortable with the SUD screening process, 6 themes emerged for both adolescents and caregivers. Both groups talked about issues with “Privacy and confidentiality,” “Clear communication and transparency,” and “Supportive and non-judgmental environments.” Adolescents discussed themes of having a “Supportive presence,” “Comfortable environment,” and feelings of “Respect and Empowerment.” Caregivers discussed “Involvement and consent,” “Faster and less intrusive processes,” and “Assurance of accurate testing methods.”

When asked for suggestions on improvements to the screening process, 9 distinct themes emerged. Both groups discussed issues of “Privacy and confidentiality,” “Clear communication and transparency,” “Non-judgmental and supportive environment,” “Faster and less intrusive process,” and “Follow-up support and resources.” Adolescents discussed themes of a “Comfortable environment” and “Respect and empowerment,” whereas caregivers touched on “Involvement and consent” and “Cultural sensitivity and personalization.”

When asked how doctors can better support adolescents during drug screening, 9 distinct themes emerged. Both adolescents and caregivers mentioned “Privacy and confidentiality,” Clear communication and transparency,” “Non-judgmental and supportive environment,” and “Follow-up support and resources.” Adolescents discussed themes of “Respect and empowerment” and “Involving parents thoughtfully,” whereas caregivers talked about “Involvement and consent,” “Use of age-appropriate language and tools,” and “Normalize screening and reduce stigma.”

## 4 Limitations

Several limitations should be acknowledged. First, the study samples may not represent the U.S. adolescent or caregiver populations. This study was conducted via an online national survey to capture generalizable perceptions of substance use screening rather than experiences confined to a single ED setting. Although this approach expands the study’s reach, it limits direct comparison with the demographic profile of pediatric ED patients. Future studies should compare attitudes toward screening between general adolescent populations and those receiving ED care.

The lack of accounting for individual experiences with substances is a limitation because users and nonusers may hold different perspectives. Recruitment through an online survey platform can introduce a selection bias, favoring participants with consistent internet access and a willingness to engage in online surveys. Additionally, the topic of our questions may be subject to social desirability bias, particularly in sensitive topics, such as substance use. Finally, although qualitative data enriched the findings, the open-ended responses were analyzed post hoc and may lack the depth achievable through formal mixed methods designs.

The proportion of respondents with prior ED was higher than national estimates. Although prior ED experience was not a selection criterion, individuals with health care experience may have been more likely to participate, potentially introducing selection bias. Future studies could aim to recruit a more representative sample or compare perceptions between those with and without prior ED visits.

The study’s response rate was low (17% adolescents, 15.4% caregivers), which may introduce selection bias and limit generalizability. Respondents willing to participate in an online survey about substance use screening may hold different views than nonrespondents.

Generalizability of our findings is likely relevant to a wide range of pediatric health care settings and adolescent and caregiver populations in the United States, based on the demographic diversity of our sample. However, the variability in regional and cultural attitudes toward substance use, SUD treatment, and health care, as well as differences in the legal and regulatory landscape across the states and local standards, may limit the generalizability of these findings in all regions or settings.

## 5 Discussion

A significant proportion of adolescents (43.8%) and caregivers (42.6%) supported universal substance use screening in the ED. The findings highlight that ensuring privacy, confidentiality, and a nonjudgmental approach—while addressing adolescents’ need for autonomy and caregivers’ concerns about transparency and involvement—are critical for optimizing the acceptability of SUD screening. These results emphasize the necessity of tailoring screening programs to balance the priorities of both adolescents and caregivers while fostering trust and engagement in care.

Although demographic data were collected, subgroup analysis of how demographics influenced screening perceptions did not show any significant differences except for perceived trust in health care staff. Future research should explore whether factors such as socioeconomic status, prior ED experience, and racial/ethnic background shape attitudes toward substance use screening.

A key finding was the significant concern about potential legal consequences associated with screening. Given the punitive approach to substance use in many states and the stigma that fuels this fear, apprehension is understandable. However, these fears may deter adolescents from engaging in screening and subsequent intervention. Addressing legal concerns through clear communication about confidentiality protections—and tailoring screening protocols to align with specific state regulations—is essential. Some states mandate reporting of substance use under specific circumstances, such as during pregnancy or in cases of suspected child endangerment. Therefore, ED-based screening programs may require state-specific adaptations to ensure alignment with local legal frameworks while reinforcing the goal of supportive, nonpunitive care.

Beyond legal concerns, a notable finding was the lack of trust in health care staff. This reflects broader trends in declining societal trust in institutions, including medicine. Although health care professionals remain among the most trusted experts, according to recent Pew Research Center data, skepticism persists, particularly among marginalized communities.^23^ Previous research on physician−patient trust suggests that demographic concordance and physician communication style significantly influence perceived trustworthiness.^24^ In the ED setting, where interactions are brief and often high-stakes, cultivating trust is challenging but vital.^25^ Further research is needed to explore how ED staff can enhance trust—potentially through transparency, shared decision-making, and improved staff−patient communication.

Despite concerns about privacy and legal consequences, adolescents in our study demonstrated a clear interest in speaking with professionals about substance use. This aligns with prior studies suggesting that the ED may serve as an underutilized setting for brief interventions and referrals for adolescent substance use.^15^^,^^16^ Given the limited time available during ED visits, future research should explore innovative strategies—such as digital screening tools, telehealth follow-ups, or embedded behavioral health specialists—to facilitate these conversations while addressing adolescent and caregiver concerns.

Notably, our study found lower rates of perceived importance for SUD screening compared to previous research. For example, O’Mara et al^11^ reported that 63% of adolescents and 68% of caregivers viewed SUD screening as important, whereas in our study, these rates were 50.4% and 38.0%, respectively. Similarly, Langerman et al^10^ found higher rates of support for universal screening among adolescents (73%) and caregivers (84%). These differences may be attributable to variations in study populations, data collection settings, or the increasing societal focus on privacy and autonomy in health care interactions. Prior studies recruited participants from EDs immediately following clinical encounters, whereas our study used an online national panel, which may have included individuals with different levels of health care engagement. Although direct comparisons should be made cautiously, these variations underscore the evolving landscape of adolescent and caregiver attitudes toward SUD screening.

Renny et al^13^ found that 71.8% of adolescents thought it was important to ask about drug and alcohol use in the ED, a notably higher proportion than the 50.4% in our study who rated screening as “very” or “extremely” important. This difference may be attributed to the timing of the survey; adolescents in Renny’s study were surveyed in the ED setting, where acute health care concerns might heighten perceptions of the importance of screening, whereas our study participants completed surveys in a nonclinical context.

Future studies should explore adolescents' and caregivers' concerns about the reporting of potentially illegal behaviors during SUD screening and identify what assurances would mitigate these fears. Investigating how confidentiality policies and legal protections influence willingness to disclose substance use could inform more effective screening approaches. Additionally, research should assess the impact of different messaging strategies on building trust and reassuring both teens and parents about the screening process.

Our findings suggest that substance use screening in pediatric EDs was supported by a substantial proportion of adolescents and caregivers but remains a topic requiring further exploration. Both adolescents and caregivers express concerns such as fear of confidentiality breach, judgment, stigma, and legal consequences, highlighting the need for tailored, confidential, and respectful approaches to optimize engagement and effectiveness.

## Author Contributions

SS, CC, and AEZ conceptualized and planned the study. SS, CC, CB, THC, and AEZ developed and validated the survey. HSP supported the thematic analysis and qualitative results. SS drafted the manuscript, and all authors contributed substantially to its revision. SS takes responsibility for the paper as a whole.

## Funding and Support

This study was funded by the Penn State College of Medicine Junior Faculty Development Program Grant (SS). The funding organization had no role in the design, implementation, or analysis of the research or the preparation of this manuscript. This work was partially funded in part by 10.13039/100000102HRSA (U03MC49671) through the PECARN SPARC node (THC). The Adolescent Health Network detailed in the manuscript methods is currently supported by the 10.13039/100006108National Center for Advancing Translational Sciences, National Institutes of Health, through Grant UL1 TR002014. The content is solely the responsibility of the authors and does not necessarily represent the official views of the NIH.

## Conflict of Interest

AEZ is a member of the Board of Directors and Vice President of the American Society of Addiction Medicine. Other authors declare no conflicts of interest relevant to this study.
