# Supplementary material for: A Survey of Adolescent and Caregiver Perceptions of Substance Use Screening in Pediatric Emergency Departments
Source: J Am Coll Emerg Physicians Open. 2025 Jun 7;6(4):100198. doi: 10.1016/j.acepjo.2025.100198 (PMC12174557; doi:10.1016/j.acepjo.2025.100198)
Supplement: Supplementary Material [file mmc1.docx]

**Supplementary Material – Surveys for Caregivers and Adolescents:**

**Caregiver Survey for Substance Use Screening**

Demographics

1. What is your age?
   1. 20-29
   2. 30-39
   3. 40-49
   4. 50-59
   5. 60-69
   6. 70-79
2. What is your child's age?
   1. 13
   2. 14
   3. 15
   4. 16
   5. 17
3. What is your gender?
   1. Male
   2. Female
   3. Non-binary
   4. Genderqueer
   5. Genderfluid
   6. Prefer not to say
   7. Prefer to self-describe _______
4. What is your ethnicity?
   1. White/Caucasian
   2. Black/African American
   3. Hispanic/Latino/Latina/Latinx
   4. Asian
   5. Native American/Alaska Native
   6. Native Hawaiian/Pacific Islander
   7. Middle Eastern/North African
   8. Multiracial/Multiethnic
   9. Prefer not to say
   10. Prefer to self-describe____________
5. What is your highest level of education completed?
   1. Less than high school
   2. High school graduate
   3. Some college
   4. 2 year degree
   5. 4 year degree
   6. Professional degree
   7. Doctorate
6. Have you ever visited the emergency department for your child before?
   1. Yes
   2. No
   3. Not sure
7. How many times have you visited the emergency department for your child?
   1. 1
   2. 2-3
   3. 4 or more

- Display This Question:
  - If Have you ever visited the emergency department for your child before? = Yes
  - Or Have you ever visited the emergency department for your child before? = Not sure

Substance Use Screening Experience

1. Has your child ever been screened for drug use in the emergency department?
   1. No
   2. Maybe
   3. Yes

- Display This Question:
  - If Have you ever visited the emergency department for your child before? = Yes
  - Or Have you ever visited the emergency department for your child before? = Not sure

1. How many times has your child been screened for drug use in the emergency department?
   1. Never
   2. Once
   3. More than once
   4. Not sure

- Display This Question:
  - If Have you ever visited the emergency department for your child before? = Yes
  - Or Have you ever visited the emergency department for your child before? = Not sure

1. Have you ever been present during your child’s drug use screening in the emergency department?
   1. Yes
   2. No
   3. Not sure
2. How comfortable do you think your child felt during the screening process?
   1. Extremely uncomfortable
   2. Somewhat uncomfortable
   3. Neither comfortable nor uncomfortable
   4. Somewhat comfortable
   5. Extremely comfortable

- Display This Question:
  - If Has your child ever been screened for drug use in the emergency department? = Maybe
  - Or Has your child ever been screened for drug use in the emergency department? = Yes

1. How do you feel about the length of the screening process?
   1. Extremely inappropriate
   2. Somewhat inappropriate
   3. Neither appropriate nor inappropriate
   4. Somewhat appropriate
   5. Extremely appropriate

- Display This Question:
  - If Has your child ever been screened for drug use in the emergency department? = Maybe
  - Or Has your child ever been screened for drug use in the emergency department? = Yes

1. Do you think the screening process took too much time during your emergency department visit?
   1. No
   2. Maybe
   3. Yes

- Display This Question:
  - If Has your child ever been screened for drug use in the emergency department? = Maybe
  - Or Has your child ever been screened for drug use in the emergency department? = Yes

Attitudes and Perceptions Towards Screening

1. How important do you think it is for adolescents to be screened for drug use in the emergency department?
   1. Not at all important
   2. Slightly important
   3. Moderately important
   4. Very important
   5. Extremely important
2. Do you believe screening for drug use should be a routine part of emergency department visits?
   1. Yes
   2. No
   3. Maybe
   4. Not Sure
3. Do you think drug use screening in the emergency department can help teens?
   1. Definitely not
   2. Probably not
   3. Might or might not
   4. Probably yes
   5. Definitely yes
4. How do you feel about doctors asking your child about their drug use?
   1. Extremely uncomfortable
   2. Somewhat uncomfortable
   3. Neither comfortable nor uncomfortable
   4. Somewhat comfortable
   5. Extremely comfortable
5. How comfortable are you discussing your child's drug use with healthcare providers?
   1. Extremely uncomfortable
   2. Somewhat uncomfortable
   3. Neither comfortable nor uncomfortable
   4. Somewhat comfortable
   5. Extremely comfortable
6. How likely are you to encourage your child to be honest during drug use screening?
   1. Extremely unlikely
   2. Somewhat unlikely
   3. Neither likely nor unlikely
   4. Somewhat likely
   5. Extremely likely
7. Do you think the results of your child’s screening should be shared with you?
   1. Definitely not
   2. Probably not
   3. Might or might not
   4. Probably yes
   5. Definitely yes
8. Do you think the screening results should be shared with the child's school counselor?
   1. Definitely not
   2. Probably not
   3. Might or might not
   4. Probably yes
   5. Definitely yes
9. What concerns do you have about your child being screened for drug use?
10. How much do you think the following are barriers to screening for drug use?

|  | Strongly disagree | Somewhat disagree | Neither agree nor disagree | Somewhat agree | Strongly agree |
| --- | --- | --- | --- | --- | --- |
| How long it will take | o | o | o | o | o |
| Privacy Concerns | o | o | o | o | o |
| It's not important | o | o | o | o | o |
| I worry about what other people will think | o | o | o | o | o |
| Fear of Legal Consequences | o | o | o | o | o |
| Lack of Trust in Healthcare Providers | o | o | o | o | o |
| Cultural or Religious Beliefs | o | o | o | o | o |
| Parental or Guardian Presence | o | o | o | o | o |
| Fear of Treatment or Intervention | o | o | o | o | o |
| Time Pressure | o | o | o | o | o |

1. How likely are you to seek help for your child if they have drug use issues?
   1. Extremely unlikely
   2. Somewhat unlikely
   3. Neither likely nor unlikely
   4. Somewhat likely
   5. Extremely likely
2. How likely are you to talk to a doctor about your child's drug use in the future?
   1. Extremely unlikely
   2. Somewhat unlikely
   3. Neither likely nor unlikely
   4. Somewhat likely
   5. Extremely likely
3. Do you think the emergency department is a good place to get information about substance use for your child?
   1. Definitely not
   2. Probably not
   3. Might or might not
   4. Probably yes
   5. Definitely yes
4. If your child had a positive screen in the emergency department, how useful would you find each of these?

|  | Not at all useful | Slightly useful | Moderately useful | Very useful | Extremely useful |
| --- | --- | --- | --- | --- | --- |
| Brochures on drug use | o | o | o | o | o |
| Information on where to go for more help | o | o | o | o | o |
| Speak with a professional in the Emergency Department | o | o | o | o | o |
| An app with information about drug use | o | o | o | o | o |

Suggestions for Improvement

1. What suggestions do you have to improve the substance use screening process?

_______________________________________________________________

1. How can doctors better support adolescents and their families during substance use screening?

**Adolescent Perception on Substance Use Screening**

Demographics

1. What is your age
   1. 13
   2. 14
   3. 15
   4. 16
   5. 17
2. What is your gender?
   1. Male
   2. Female
   3. Non-binary
   4. Genderqueer
   5. Genderfluid
   6. Prefer not to say
   7. Prefer to self-describe __________________________________________________
3. What is your ethnicity?
   1. White/Caucasian
   2. Black/African American
   3. Hispanic/Latino/Latina/Latinx
   4. Asian
   5. Native American/Alaska Native
   6. Native Hawaiian/Pacific Islander
   7. Middle Eastern/North African
   8. Multiracial/Multiethnic
   9. Prefer not to say
   10. Prefer to self-describe __________________________________________________
4. What is your living situation most of the time?
   1. With both parents
   2. With mother only
   3. With father only
   4. Joint custody/shared between parents
   5. With grandparents
   6. With other relatives (e.g., aunts, uncles)
   7. With foster parents
   8. In a group home
   9. In a boarding school or residential school
   10. Independent living (e.g., alone or with roommates)
   11. Prefer not to say
   12. Prefer to self-describe_________________________________________
5. Have you ever visited the emergency department before?
   1. Yes
   2. No
   3. Not sure
6. How many times have you visited the emergency department in your life?
   1. 1
   2. 2-3
   3. 4 or more

- Display This Question:
  - If Have you ever visited the emergency department before? = Yes
  - Or Have you ever visited the emergency department before? = Not sure

Substance Use Screening Experience

1. Have you ever been screened for drug use (tobacco marijuana, alcohol or other drugs) in the emergency department?
   1. Yes
   2. No
   3. Not sure

- Display This Question:
  - If Have you ever visited the emergency department before? = Yes
  - Or Have you ever visited the emergency department before? = Not sure

1. How many times have you been screened for drug use in the emergency department?
   1. Never
   2. Once
   3. More than once
   4. Not sure

- Display This Question:
  - If Have you ever been screened for drug use (tobacco marijuana, alcohol or other drugs) in the emerg... = Yes
  - Or Have you ever been screened for drug use (tobacco marijuana, alcohol or other drugs) in the emerg... = Not sure

1. Was your parent every present for your drug use screening in the emegency department?
   1. Yes
   2. No
   3. Not sure

- Display This Question:
  - If Has your child ever been screened for drug use in the emergency department? = Maybe
  - Or Has your child ever been screened for drug use in the emergency department? = Yes

1. How comfortable did you feel during the screening process?
   1. Extremely uncomfortable
   2. Somewhat uncomfortable
   3. Neither comfortable nor uncomfortable
   4. Somewhat comfortable
   5. Extremely comfortable

- Display This Question:
  - If Have you ever been screened for drug use (tobacco marijuana, alcohol or other drugs) in the emerg... = Yes
  - Or Have you ever been screened for drug use (tobacco marijuana, alcohol or other drugs) in the emerg... = Not sure

1. Did you feel the screening process was respectful?
   1. Definitely not
   2. Probably not
   3. Might or might not
   4. Probably yes
   5. Definitely yes

- Display This Question:
  - If Have you ever been screened for drug use (tobacco marijuana, alcohol or other drugs) in the emerg... = Yes
  - Or Have you ever been screened for drug use (tobacco marijuana, alcohol or other drugs) in the emerg... = Not sure

1. Were the screening questions were easy to understand?
   1. No
   2. Maybe
   3. Yes

- Display This Question:
  - If Have you ever been screened for drug use (tobacco marijuana, alcohol or other drugs) in the emerg... = Yes
  - Or Have you ever been screened for drug use (tobacco marijuana, alcohol or other drugs) in the emerg... = Not sure

1. Were there any questions you found difficult to answer?
   1. No
   2. Maybe
   3. Yes

- Display This Question:
  - If Have you ever been screened for drug use (tobacco marijuana, alcohol or other drugs) in the emerg... = Yes
  - Or Have you ever been screened for drug use (tobacco marijuana, alcohol or other drugs) in the emerg... = Not sure

1. How did you feel about the length of the screening process?
   1. Extremely inappropriate
   2. Somewhat inappropriate
   3. Neither appropriate nor inappropriate
   4. Somewhat appropriate
   5. Extremely appropriate

- Display This Question:
  - If Have you ever been screened for drug use (tobacco marijuana, alcohol or other drugs) in the emerg... = Yes
  - Or Have you ever been screened for drug use (tobacco marijuana, alcohol or other drugs) in the emerg... = Not sure

1. Do you think the screening process took too much time during your emergency department visit?
   1. Definitely not
   2. Probably not
   3. Might or might not
   4. Probably yes
   5. Definitely yes

- Display This Question:
  - If Have you ever been screened for drug use (tobacco marijuana, alcohol or other drugs) in the emerg... = Yes
  - Or Have you ever been screened for drug use (tobacco marijuana, alcohol or other drugs) in the emerg... = Not sure

1. Did the screening process help you understand the risks of substance use better?
   1. Definitely not
   2. Probably not
   3. Might or might not
   4. Probably yes
   5. Definitely yes

- Display This Question:
  - If Have you ever been screened for drug use (tobacco marijuana, alcohol or other drugs) in the emerg... = Yes
  - Or Have you ever been screened for drug use (tobacco marijuana, alcohol or other drugs) in the emerg... = Not sure

Attitudes and Perceptions Towards Screening

1. How important do you think it is to screen for drug use in the emergency department?
   1. Not at all important
   2. Slightly important
   3. Moderately important
   4. Very important
   5. Extremely important
2. Do you believe screening for drug use should be a routine part of emergency department visits?
   1. Yes
   2. Maybe
   3. No
   4. Not Sure
3. Do you think drug use screening in the emergency department can help teens?
   1. Definitely not
   2. Probably not
   3. Might or might not
   4. Probably yes
   5. Definitely yes
4. Do you think screening for drug use can help prevent drug use in your friends?
   1. Definitely not
   2. Probably not
   3. Might or might not
   4. Probably yes
   5. Definitely yes
5. How do you feel about being asked questions about drug use?
   1. Extremely uncomfortable
   2. Somewhat uncomfortable
   3. Neither comfortable nor uncomfortable
   4. Somewhat comfortable
   5. Extremely comfortable
6. How comfortable would you be about discussing your drug use with doctors in the Emergency Department?
   1. Extremely uncomfortable
   2. Somewhat uncomfortable
   3. Neither comfortable nor uncomfortable
   4. Somewhat comfortable
   5. Extremely comfortable
7. How likely are you to be honest about your drug use in the Emergency Department?
   1. Extremely unlikely
   2. Somewhat unlikely
   3. Neither likely nor unlikely
   4. Somewhat likely
   5. Extremely likely
8. Do you think the results of the screening should be shared with your parents/guardians?
   1. Definitely not
   2. Probably not
   3. Might or might not
   4. Probably yes
   5. Definitely yes
9. Do you think the results of the screening should be shared with your school counselor?
   1. Definitely not
   2. Probably not
   3. Might or might not
   4. Probably yes
   5. Definitely yes
10. What concerns do you have about being screened for substance use?

________________________________________________________________

Barriers to Screening

1. What would make you feel more comfortable during the screening process?

________________________________________________________________

1. How much do you think the following are barriers to screening for drug use?

|  | Strongly disagree | Somewhat disagree | Neither agree nor disagree | Somewhat agree | Strongly agree |
| --- | --- | --- | --- | --- | --- |
| How long it will take | o | o | o | o | o |
| Privacy Concerns | o | o | o | o | o |
| It's not important | o | o | o | o | o |
| I worry about what other people will think | o | o | o | o | o |
| Fear of Legal Consequences | o | o | o | o | o |
| Lack of Trust in Healthcare Providers | o | o | o | o | o |
| Cultural or Religious Beliefs | o | o | o | o | o |
| Parental or Guardian Presence | o | o | o | o | o |
| Fear of Treatment or Intervention | o | o | o | o | o |
| Time Pressure | o | o | o | o | o |

1. How likely are you to seek help if you have substance use issues?
   1. Extremely unlikely
   2. Somewhat unlikely
   3. Neither likely nor unlikely
   4. Somewhat likely
   5. Extremely likely
2. How likely are you to talk to a healthcare provider about substance use in the future?
   1. Extremely unlikely
   2. Somewhat unlikely
   3. Neither likely nor unlikely
   4. Somewhat likely
   5. Extremely likely
3. Do you think the emergency department is a good place to get information about substance use?
   1. Definitely not
   2. Probably not
   3. Might or might not
   4. Probably yes
   5. Definitely yes
4. If you had a positive screen in the emergency department, how useful would you find each of these?

|  | Not at all useful | Slightly useful | Moderately useful | Very useful | Extremely useful |
| --- | --- | --- | --- | --- | --- |
| Brochures on drug use | o | o | o | o | o |
| Information on where to go for more help | o | o | o | o | o |
| Speak with a professional in the Emergency Department | o | o | o | o | o |
| An app with information about drug use | o | o | o | o | o |

1. What suggestions do you have to make drug use screening a better process for you?

________________________________________________________________

1. How can doctors better support adolescents during drug use screening?

_______________________________________________________________
